# Perioperative management of anticoagulant therapy

**DOI:** 10.1515/iss-2019-0004

**Published:** 2019-07-18

**Authors:** Johanna Wagner, Johan F. Lock, Carolin Kastner, Ingo Klein, Katica Krajinovic, Stefan Löb, Christoph-Thomas Germer, Armin Wiegering

**Affiliations:** Department of General, Visceral, Transplantation, Vascular and Pediatric Surgery, Medical Center Julius-Maximilians, University of Wuerzburg, Oberduerrbacherstr. 6, 97080 Wuerzburg, Germany, Phone: +49931 20131001; Department of General, Visceral, Transplantation, Vascular and Pediatric Surgery, University Hospital Würzburg, Wüerzburg, Germany; Comprehensive Cancer Center Mainfranken, Würzburg, Germany; Department of Biochemistry and Molecular Biology, University of Würzburg, Würzburg, Germany

**Keywords:** anticoagulation, bridging, dalteparin, enoxaparin, NOAC, perioperative period, warfarin

## Abstract

About 10% of patients taking a chronic, oral anticoagulant therapy require an invasive procedure that can be associated with an increased risk for peri-interventional or perioperative bleeding. Depending on the risk for thromboembolism and the risk for bleeding, the physician has to decide whether the anticoagulant therapy should be interrupted or continued. Patient characteristics such as age, renal function and drug interactions must be considered. The perioperative handling of the oral anticoagulant therapy differs according to the periprocedural bleeding risk. Patients requiring a procedure with a minor risk for bleeding do not need to pause their anticoagulant therapy. For procedures with an increased risk for perioperative bleeding, the anticoagulant therapy should be adequately paused. For patients on a coumarin derivative with a high risk for a thromboembolic event, a perioperative bridging therapy with a low molecular weight heparin is recommended. Due to an increased risk for perioperative bleeding in patients on a bridging therapy, it is not recommended in patients with a low risk for thromboembolism. For patients taking a non-vitamin K oral anticoagulant, a bridging therapy is not recommended due to the fast onset and offset of the medication.

## Introduction

An increasing number of patients receive long-term anticoagulation with phenprocoumon, warfarin, or one of the novel direct oral anticoagulants. About 10% of these patients per year require a surgery or an invasive procedure and therefore an interruption of their anticoagulation [1]. The most common indication for an anticoagulant therapy is atrial fibrillation as the anticoagulant therapy can reduce the risk for an embolic event, especially for stroke, by up to 60%. Atrial fibrillation has a prevalence of 3% in the western world [2], with an increasing prevalence over time [3], [4]. However, these patients only have, on average, a 2–4% risk for an embolic event per year [5]. The risk for thromboembolism is elevated in patients with a higher CHA_2_DS_2_-VASc score (see Tables 1 and 2). Other indications for an anticoagulation therapy are, for example, patients after thrombo-embolic events (pulmonary embolism, deep vein thrombosis (DVT)), valvular transplant or patients with thrombophilia. In these cases, the risk for an embolic event is usually higher and the benefit of an anticoagulant therapy larger.

**Table 1:** CHA_2_DS_2_-VASc score.

Acronym	Risk factor	Score
C	Congestive heart failure	1
H	Hypertension	1
A_2_	Age ≥75 years	2
D	Diabetes mellitus	1
S_2_	Stroke/TIA/thromboembolism	2
V	Vascular disease	1
A	Age 65–74 years	1
Sc	Sex category: female sex	1

**Table 2:** Adjusted stroke rate according to the CHA_2_DS_2_-VASc score [6].

Score	Adjusted stroke rate (% per year)
0	0
1	1.3
2	2.2
3	3.2
4	4.0
5	6.7
6	9.8
7	9.6
8	6.7
9	15.2

In planning an elective surgery, the surgeon must address the question of whether the anticoagulant therapy should be paused, continued, or bridged, for example with heparin. For this decision multiple factors are important, such as patient characteristics (renal function, indication for anticoagulant therapy, age, patient history of bleeding or thromboembolic complications) and surgical factors (especially the perioperative bleeding risk).

## Available anticoagulant medication

For patients with an indication for long-term anticoagulation therapy, two orally administered medication groups exist: coumarin anticoagulants and non-vitamin K oral anticoagulants (NOACs).

### Coumarin derivatives

Phenprocoumon and warfarin are coumarin derivatives. They are vitamin K antagonists that inhibit the synthesis of vitamin K-dependent coagulation factors. The thromboplastin time with the international normalized ratio (INR) measures the effect of phenprocoumon and warfarin. Due to intraindividual variability in the dose-response, frequent monitoring of the INR is necessary. The needed dose is taken once daily. For most indications an INR of 2–3 is sufficient, whereas, for example, in patients with prosthetic heart valves, a higher INR is recommended [7]. When beginning a phenprocoumon or warfarin therapy, a loading dose is sensible to reach the desired INR. This is usually achieved within 3–7 days after the begin of the therapy. Phenprocoumon and warfarin bind to albumin in the serum, leading to a reduced effectiveness by hypoalbuminemia.

### NOACs

This newer group of orally administered anticoagulants displays multiple advantages over the coumarin derivatives. Due to more predictable pharmacokinetics, few drug interactions and a rapid onset and offset, regular monitoring is not necessary. This is often a great relief for patients on chronic anticoagulant therapy.

#### Dabigatran elixate

Dabigatran elixate is a prodrug that is metabolized into dabigatran. Dabigatran is a selective and reversible thrombin inhibitor that has low bioavailability (3–7%), and about 80% are renally eliminated. Thus, in patients with renal insufficiency the half-life is prolonged from 12–17 h up to 24 h. In case of severe renal insufficiency (creatinine clearance <30 mL/min/1.73 m^2^), dabigatran is contraindicated in Europe, whereas, in North America, reduced doses are recommended [8]. The normal, recommended dosage is 110 or 150 mg twice daily [9]. The effectiveness of dabigatran can be measured by the ecarin-coagulation time.

#### Rivaroxaban

Rivaroxaban is a selective and reversible direct factor Xa inhibitor. It has a half-life of 5–9 h, which is prolonged to 11–13 h in elderly patients above 75 years of age. The bioavailability is 80–100%. Rivaroxaban is mostly (2/3) metabolized in the liver; thus, the elimination is only mildly dependent on the renal function. However, in patients with a creatinine clearance of <15 mL/min/1.73 m^2^, rivaroxaban is contraindicated [8], [10]. The standard dosing is 20 mg once daily with a recommended reduction to 15 mg/day in patients with a reduced creatinine clearance of 15–49 mL/min/1.73 m^2^ [9]. The factor Xa activity measures the effectiveness of the rivaroxaban therapy.

#### Apixaban

Apixaban is also a selective and reversible direct factor Xa inhibitor with a longer half-life of 8–15 h. The bioavailability is 50%, and like rivaroxaban, apixaban is metabolized to 2/3 in the liver. Also, in patients with a creatinine clearance of <15 mL/min/1.73 m^2^, apixaban is contraindicated. Apixaban (5 mg) is administered twice daily. This dose is reduced to 2.5 mg twice daily in patients with severe chronic kidney disease [9]. The effectiveness of apixaban therapy is also measured by the factor Xa activity [8].

#### Edoxaban

Edoxaban is a relatively new reversible factor Xa inhibitor that was approved by the Food and Drug Administration (FDA) in 2015. The half-life is 9–11 h and the bioavailability 62%. Due to 50% renal elimination, a dose reduction is recommended in patients with renal insufficiency, and in patients with a creatinine clearance of <15 mL/min/1.73 m^2^, edoxaban is contraindicated [11]. The standard dosing is 60 mg once daily and is reduced to 30 mg/day in patients with renal insufficiency [9]. The effectiveness can also be measured by the factor Xa activity [12].

For all NOACs, plasma concentration can be used to verify if the desired therapeutic window has been reached and allows to estimate the current level of anticoagulation. Table 3 gives an overview of the expected plasma concentrations in a therapeutic use.

**Table 3:** Peak plasma concentration levels to be expected after therapeutic doses of dabigatran, rivaroxaban, apixaban or edoxaban [13].

Anticoagulant drug	Peak plasma concentration (ng/mL)	
	Nonvalvular atrial fibrillation	Venous thromboembolism
Dabigatran	117–275	117–275
Rivaroxaban	184–343	189–419
Apixaban	91–321	59–302
Edoxaban	125–245	149–317

## Perioperative handling

When the surgeon must decide the perioperative handling of the patient’s anticoagulant therapy, there are multiple factors to be considered, such as the indication of anticoagulant therapy, the risk for an embolic event and the risk for perioperative bleeding. In general, there are three different options concerning the perioperative handling of an anticoagulation therapy:

1. Continuing the oral anticoagulation therapy
2. Perioperative pause
3. Perioperative bridging with heparin

### Continuing the oral anticoagulation therapy

Pausing or changing the anticoagulation therapy can lead to a temporary over- or undercoagulation, which could have severe consequences, such as embolic events or severe bleeding. Thus, a perioperative pause should carefully be considered. Patients undergoing operations with a low risk for bleeding should continue their oral anticoagulation therapy (see Table 4). The INR in patients taking warfarin can be left in the therapeutic range. NOACs do not need to be paused either, but – if possible – the operation should be conducted in the time of the lowest plasma levels (i.e. >12 or 24 h after the last administration) [14], [15], [16].

**Table 4:** Classification of surgical procedures according to the perioperative bleeding risk [14].

Minor bleeding risk	Dental procedures (abscess incision, extraction of one to three teeth, paradontal surgery, implant positioning) Cataract or glaucoma intervention Endoscopy without biopsy Superficial surgery (abscess incision, dermatologic excisions, etc.)
Low bleeding risk	Endoscopy with biopsy Prostate or bladder surgery Catheter ablation Angiography (noncoronary) Pacemaker or ICD implantation Diagnostic and simple laparoscopy Superficial lymph node excision Thoracentesis or paracentesis Port catheter implantation Open inguinal hernia operation and umbilical hernia operations
High bleeding risk	Complex endoscopy Spinal or epidural anesthesia Lumbar puncture Thoracic surgery Abdominal surgery Major orthopedic and trauma surgery Liver and kidney biopsy Urologic surgery Extracorporeal shockwave lithotripsy Cardiac, intracranial, or spinal surgery Vascular surgery
High bleeding risk and high thromboembolic risk	Complex left-sided ablation

### Perioperative pause

The indication for the anticoagulant therapy should be carefully discussed. Many patients receive an anticoagulant therapy although the indication is not clear. In these cases, the anticoagulant therapy should not be continued or bridged during the perioperative period due to an increased bleeding risk.

#### Coumarin derivatives

In operations with an increased risk for bleeding, whether slightly, moderately, or highly increased, coumarin derivatives should be paused during the perioperative period. To reach a normalized INR, the preoperative pause should be at least 7 days and usually should be prolonged to 10 days in elderly patients. Vitamin K (10 mg) administered orally can help normalize the INR within 24 h [17]. An intravenous administration of vitamin K is not recommended as it can lead to an increased risk for thromboembolism. Prothrombin complex concentrate is an effective, fast working and controllable antidote that can be used prior to emergency operations.

Depending on the perioperative risk for thromboembolism, either a thrombosis prophylaxis or a bridging with low molecular weight heparin (LMWH) is recommended after reaching a normalized INR.

Postoperatively, the restart of the anticoagulant therapy depends on the bleeding risk. Patients can start taking warfarin as early as the first postoperative day, because a therapeutic effect of the coumarin derivatives is only reached after 4–7 days [18]. Due to the oral administration, patients after abdominal surgery should wait for a normalized gastrointestinal passage before retaking the oral anticoagulant medication. Until the coumarin derivatives have reached their therapeutic effect either thromboembolism prophylaxis or a continuation of the bridging therapy is recommended.

#### NOACs

The perioperative handling of NOACs is considerably easier than that of warfarin due to the fast onset and offset. For operations with an increased risk for bleeding, NOACs should be preoperatively paused. The duration of the pause depends mainly on the patient’s renal function and the risk for perioperative bleeding (see Tables 4 and 5) [8], [19], [20], [21]. Certain medications prolong the half-life of NOACs, such as amiodarone and dronedarone, acetylsalicylic acid, nonsteroidal anti-inflammatory drugs, phenothiazine, verapamil, diltiazem, ketoconazole, fluconazole, macrolides, and HIV protease inhibitors. In case of a co-medication with NOACs, the preoperative pause should be prolonged by 12 h [14], [19]. A bridging with heparin preoperatively is not necessary. Information regarding the restart of NOACs postoperatively only scarcely exists due to the quick effectiveness of NOACs. Thus, the restart of an NOAC medication should be when the risk for postoperative bleeding is low and the gastrointestinal passage in normalized. After small operations with low risk for bleeding, NOACs can be continued earliest 6–8 h and latest 24 h postoperatively [14], [19], [21]. After operations with a higher risk for bleeding, NOACs should be restarted earliest 48–72 h postoperatively [14].

**Table 5:** Duration of preoperative pause between last NOAC intake and the planned operation [14].

	Creatinine clearance (mL/min/1.73 m^2^)			
	≥80	50–80	30–50	15–30
Operations with a low risk for bleeding				
Dabigatran	≥24	≥36	≥48	Not indicated
Rivaroxaban	≥24	≥24	≥24	≥36
Edoxaban	≥24	≥24	≥24	≥36
Abixaban	≥24	≥24	≥24	≥36
Operations with a high risk for bleeding				
Dabigatran	≥48	≥72	≥96	Not indicated
Rivaroxaban	≥48	≥48	≥48	≥48
Edoxaban	≥48	≥48	≥48	≥48
Abixaban	≥48	≥48	≥48	≥48

If the surgical procedure is urgent and the perioperative risk for bleeding high, the administration of an antidote should be considered [22], [23]. Before administering an antidote, the level of anticoagulation should be determined. The measurement of the plasma level is recommended as it reflects the amount of medication still present [13]. Idarucizumab is an antidote for dabigatran and is approved for the usage before emergency surgeries with a high bleeding risk when the plasma level is above 30 ng/mL. In the event of a major bleeding, idarucizumab may be given when dabigatran plasma levels are over 50 ng/mL. Idarucizumab binds to dabigatran and its metabolites with high affinity and renally eliminates these complexes [22], [24]. Two doses of 2.5 mg idarucizumab each are administered intravenously over a short time course of 15 min [22], [23]. For the factor Xa inhibitors rivaroxaban and apixaban, andexanet alpha was recently approved as an antidote in major bleeding events. However, it is not yet approved for a preoperative use before emergency surgery. Andexanet alpha binds to rivaroxaban and apixaban and eliminates these substances [22], [25]. In bleeding events, it is administered intravenously with a bolus followed by a continuous intravenous infusion over 120 min (low-dose regimen: 400 mg bolus+4 mg/min infusion versus high-dose regimen: 800 mg bolus+8 mg/min infusion) [25], [26]. The type of regimen is based on the dose of anticoagulation and the time interval since the last intake [26].

### Perioperative bridging with heparin

For patients with a moderate to high risk for thromboembolsim as well as a risk for perioperative bleeding when pausing the oral anticoagulation, a bridging therapy with heparin should be considered (see Table 6). If patients are only temporarily on oral anticoagulation, the surgeon should consider postponing the surgery if possible until the anticoagulation therapy is completed. Usually, LMWH is used for the perioperative bridging therapy. Interestingly, these substances are not approved for this indication. LMWH can be used as a full-dose anticoagulation, which equals an INR of 2.5–3.2 or as a half-dose anticoagulation, equaling an INR of about 2.0. In patients with renal insufficiency and a clear indication for perioperative bridging (i.e. patients after mitral valve replacement), the intravenous administration of unfractionated heparin is an alternative to LMWH [27].

**Table 6:** Thromboembolic risk stratification [14].

Low	Medium	High
CHA_2_DS_2_-VASc 0–2 DVT >12 months Double wing aortic valve >3 months with sinus rhythm	CHA_2_DS_2_-VASc 3–4 DVT 3–12 months DVT in cancer patients Recurring DVT Double wing aortic valve with CHA_2_DS_2_-VASc>0 Biological heart valve with sinus rhythm	CHA_2_DS_2_-VASc 5–6 DVT <3 months DVT with pulmonary embolism <12 months Stroke <3 months Mitral valve replacement Older mechanical aortic valves Biological heart valve with atrial fibrillation Hereditary thrombophilias

Due to an increased perioperative bleeding risk in patients receiving a perioperative bridging therapy, a bridging therapy should be carefully considered. A meta-analysis reviewed 34 studies assessing the perioperative bleeding risk in patients receiving a bridging therapy. Patients with a perioperative bridging therapy with LMWH had an increased risk for overall and major bleeding events [overall bleeding risk: odds ratio, 5.40; 95% confidence interval (CI), 0.42–1.54 and major bleeding risk: odds ratio, 3.60; 95% CI, 1.52–8.50]. This meta-analysis also looked at the rate of a thromboembolic event in patients receiving and not receiving a perioperative bridging therapy and found no difference in the occurrence rate. Increasing the bridging therapy from a half-dose to a full-dose anticoagulation increases the overall bleeding risk (odds ratio, 2.28; 95% CI, 1.27–4.08) [28]. An American register analysis showed an increased bleeding risk and rate of adverse events in patients receiving a bridging therapy [29]. One prospective, randomized trial looked at the risk for a perioperative thromboembolism and the bleeding risk in patients with atrial fibrillation, who either received a perioperative bridging therapy with dalteparin or a placebo. The results showed a similar risk for thromboembolism in both groups (0.4% in the placebo group vs. 0.3% in the bridging group) with an increased risk for major bleeding and minor bleeding in the bridging group (1.3% vs. 3.2%; p=0.005 and 12.0% vs. 20.9%; p<0.001) [30]. Thus, routine bridging is not recommended, although patients with a high risk for thromboembolism do profit from a perioperative bridging therapy.

#### Bridging options

Patients with a low risk for a thromboembolic event should receive a normal thromboembolism prophylaxis and no therapeutic anticoagulation in the perioperative period (i.e. 40 mg/day enoxaparin). For patients with a medium risk for a thromboembolic event, a half-dose anticoagulation can be prescribed if the risk for perioperative bleeding is not high (i.e. 1 mg/kg/day enoxaparin). In cases of high perioperative bleeding risk and medium risk for a thromboembolic event, a normal thromboembolism prophylaxis is often the safer choice. A full-dose anticoagulation is only recommended in patients with a high risk for a thromboembolic event (see Table 7).

**Table 7:** Bridging options for patients on coumarin derivatives paused for the perioperative period.

Risk for thromboembolism	Risk for perioperative bleeding		
	Low risk	Medium risk	High risk
Low	Thromboembolism prophylaxis	Thromboembolism prophylaxis	Thromboembolism prophylaxis
Medium	Half-dose anticoagulation	Half-dose anticoagulation	Thromboembolism prophylaxis
High	Full-dose anticoagulation	Full-dose anticoagulation	Full-dose anticoagulation

#### Bridging dose

The dosage of the LMWH must be adapted according to different patient characteristics. Patients with a severe renal insufficiency [glomerular filtration rate (GFR) <15 mL/min/1.73 m^2^] should not receive LMWH due to accumulation. A 50% reduced dose should be considered in patients with a GFR of <60 mL/min/1.73 m^2^ and should be applied in patients with a GFR of 15–30 mL/min/1.73 m^2^ [31]. Elderly patients over 75 years of age should receive 75% of the normal dose [32] (see Tables 8 and 9). In obese patients with a body mass index of >40 kg/m^2^, a higher dose of LMWH should be considered [35].

**Table 8:** Bridging with enoxaparin [31].

Type of anticoagulation therapy	Dosage of enoxaparin (subcutaneous application)		
	Normal dosage	Patients with renal insufficiency (GFR 15–30 mL/min/1.73 m^2^)	Elderly patients >75 years
Thromboembolism prophylaxis	4000 IE/day (40 mg)	2000 IE/day (20 mg)	No adjustment
Half-dose anticoagulation	1000 IE (1 mg)/kg/day or 4000 IE (40 mg) twice daily	500 IE (0.5 mg)/kg/day	750 IE (0.75 mg)/kg/day
Full-dose anticoagulation	1000 IE (1 mg)/kg twice daily	1000 IE (1 mg)/kg/day	750 IE (0.75 mg)/kg body weight twice daily

**Table 9:** Bridging with dalteparin [31], [33], [34].

Type of anticoagulation therapy	Dosage of dalteparin (subcutaneous application)		
	Normal dosage	Patients with renal insufficiency (GFR 15–30 mL/min/1.73 m^2^)	Elderly patients >75 years
Thromboembolism prophylaxis	5000 IE/day	No adjustment	No adjustment
Half-dose anticoagulation	5000 IE twice daily	5000 IE/day	No adjustment
Full-dose anticoagulation	100 IE/kg twice daily	Adjustment according to anti-factor Xa level	No adjustment

## Conclusion

In deciding the perioperative handling of an anticoagulant therapy, the patient’s risk for thromboembolism and the perioperative risk for bleeding play an important role. Anticoagulant therapy can be continued for procedures with a minor bleeding risk. If possible, the procedure should be conducted 12–24 h after the last NOAC intake. For operations with a low or high bleeding risk, the anticoagulant therapy should be paused. For NOACs, a pause of at least 24 h and up to 96 h is recommended depending on the bleeding risk and the patient’s renal function. Patients taking phenprocoumon or warfarin should stop taking the medication 7–10 days before surgery with an increased risk for bleeding. For patients with an increased risk for thromboembolism, a bridging therapy is recommended (see helpful checklist in German: supplementary material in Lock et al. [36]).

## Supplementary Material

The article (https://doi.org/10.1515/iss-2019-0004) offers reviewer assessments as supplementary material.
