# Supplementary material for: Perioperative management of anticoagulant therapy
Source: Innov Surg Sci. 2019 Jul 18;4(4):144–51. doi: 10.1515/iss-2019-0004 (PMC8059348; doi:10.1515/iss-2019-0004)
Supplement: Supplementary file 1 [file iss-04-20190004-s001.pdf]

Reviewer Assessment

Johanna Wagner*, Johan F. Lock, Carolin Kastner, Ingo Klein, Katica Krajinovic, Stefan Löb, Christoph-Thomas Germer and Armin Wiegering

Perioperative management of anticoagulant therapy

https://doi.org/10.1515/iss-2019-0004
Received March 22, 2019; accepted June 24, 2019

***Corresponding author: Johanna Wagner,** Department of General, Visceral, Transplantation, Vascular and Pediatric Surgery, Medical Center Julius-Maximilians, University of Wuerzburg, Oberduerrbacherstr. 6, 97080 Wuerzburg, Germany, Phone: +49931 20131001, E-mail: Wagner_J6@ukw.de. https://orcid.org/0000-0002-2999-6900

Reviewers' Comments to Original Submission

Reviewer 1: anonymously

May 22, 2019

**Reviewer Recommendation Term:**	Accept with Minor Revision
**Overall Reviewer Manuscript Rating:**	85
Is the subject area appropriate for you?	5 - High/Yes
Does the title clearly reflect the paper's content?	4
Does the abstract clearly reflect the paper's content?	5 - High/Yes
Do the keywords clearly reflect the paper's content?	5 - High/Yes
Does the introduction present the problem clearly?	5 - High/Yes
Are the results/conclusions justified?	4
How comprehensive and up-to-date is the subject matter presented?	3
How adequate is the data presentation?	4
Are units and terminology used correctly?	5 - High/Yes
Is the number of cases adequate?	N/A
Are the experimental methods/clinical studies adequate?	N/A
Is the length appropriate in relation to the content?	4
Does the reader get new insights from the article?	4
Please rate the practical significance.	5 - High/Yes
Please rate the accuracy of methods.	3
Please rate the statistical evaluation and quality control.	N/A
Please rate the appropriateness of the figures and tables.	4
Please rate the appropriateness of the references.	4
Please evaluate the writing style and use of language.	4
Please judge the overall scientific quality of the manuscript.	4
Are you willing to review the revision of this manuscript?	Yes

Open Access. © 2019 Wagner J., et al., published by De Gruyter. CC BY This work is licensed under the Creative Commons Attribution 4.0 Public License.

Comments to Author:

The article provides a good summary to the subject. The different anticoagulants, their characteristics and their perioperative handling are presented in a well-structured manner and yields helpful information for the clinical active surgeon.

Some questions remain about monitoring the anticoagulatory effects of these substances, especially before urgent operations. The possibility of measuring the ECT for Dabigatran and the possibility of measuring Anti-FXa activity for the other NOACs are mentioned. According to the "EHRA Practical Guide on NOACs" it is recommended to primarily use plasma concentrations rather than anti-FXa activity (Steffel et al., European Heart Journal, 2018). Some clarifications about this issue and the recommended plasma levels would be helpful.

The possibility of antagonizing oral anticoagulants is important for handling emergency situations. There are no information given on this topic. There are antidotes not only for coumarins but also for NOACs. Idarucizumab was approved for Dabgiatran Reversal (Pollack et al., N Engl J Med, 2017). In addition, Andexanet alfa was tested as reversal agent for FXa inhibitors in an open label Phase III trial (Connolly et al, N Engl J Med, 2016). The substance was eventually approved in Europe in April 2019.

Intravenous unfractioned heparin might still have a role in selected patients (e.g. patients after mitral valve replacement or with renal insufficiency). The article does not discuss this option for perioperative bridging.

We recommend to accept the paper after minor revisions.

Reviewer 2: anonymously

May 22, 2019

**Reviewer Recommendation Term:**	**Accept with Minor Revision**
**Overall Reviewer Manuscript Rating:**	**90**

Comments to Author:

This is a well written article on "Perioperative Management of Anticoagulant Therapy"

It comprises well all relevant aspects of the given task including a sound overview of the current situation, clear and concise opinions on the clinical value and gives the reader a well designed statement of the necessary action to be taken in everyday clinical practice.

The tables included allow for a quick and precise decision, the selected literature is well up to date, format and didactic sequence is of impeccable quality.

This article may well be published with minor revisions

Authors' Response to Reviewer Comments

Jun 10, 2019

We thank the reviewers for their helpful advises, which definitely improved the quality of the manuscript. Please find below a detailed point-by-point answer.

Reviewer #1: The article provides a good summary to the subject. The different anticoagulants, their characteristics and their perioperative handling are presented in a well-structured manner and yields helpful information for the clinical active surgeon. Some questions remain about monitoring the anticoagulatory effects of these substances, especially before urgent operations. The possibility of measuring the ECT for Dabigatran and the possibility of measuring Anti-FXa activity for the other NOACs are mentioned. According to the "EHRA Practical Guide on NOACs" it is recommended to primarily use plasma concentrations rather than anti-FXa activity (Steffel et al., European Heart Journal, 2018). Some clarifications about this issue and the recommended plasma levels would be helpful.

Thank you for these positive and constructive comments. We have now included the addressed points in our manuscript and have highlighted the corrections (lines 112-117; table 3).

The possibility of antagonizing oral anticoagulants is important for handling emergency situations. There are no information given on this topic. There are antidotes not only for coumarins but also for NOACs. Idarucizumab was approved for Dabgiatran Reversal (Pollack et al., N Engl J Med, 2017). In addition, Andexanet alfa was tested as reversal agent for FXa inhibitors in an open label Phase III trial (Connolly et al, N Engl J Med, 2016). The substance was eventually approved in Europe in April 2019. Intravenous unfractioned heparin might still have a role in selected patients (e.g. patients after mitral valve replacement or with renal insufficiency). The article does not discuss this option for perioperative bridging.

Thank you for addressing these important points. We have now added a section on antidots and perioperative bridging with unfractionated heparin and have highlighted the changes made. Concerning the antidot andexanet we clearly mention that it is not yet approved for preoperative administration, solely for bleeding events (lines 174-192; lines 200-202).
